# Di(2-ethylhexyl) phthalate mediates IL-33 production *via* aryl hydrocarbon receptor and is associated with childhood allergy development

**DOI:** 10.3389/fimmu.2023.1193647

**Published:** 2023-07-21

**Authors:** Mei-Lan Tsai, Shih-Hsien Hsu, Li-Ting Wang, Wei-Ting Liao, Yi-Ching Lin, Chang-Hung Kuo, Ya-Ling Hsu, Ming-Chu Feng, Fu-Chen Kuo, Chih-Hsing Hung

**Affiliations:** ^1^ Graduate Institute of Medicine, College of Medicine, Kaohsiung Medical University, Kaohsiung, Taiwan; ^2^ Department of Pediatrics, Faculty of Pediatrics, College of Medicine, Kaohsiung Medical University, Kaohsiung, Taiwan; ^3^ Department of Medical Research, Kaohsiung Medical University Hospital, Kaohsiung Medical University, Kaohsiung, Taiwan; ^4^ Research Center for Precision Environmental Medicine, Kaohsiung Medical University, Kaohsiung, Taiwan; ^5^ Department of Life Science, National Taiwan Normal University, Taipei, Taiwan; ^6^ Department of Biotechnology, College of Life Science, Kaohsiung Medical University, Kaohsiung, Taiwan; ^7^ Department of Laboratory Medicine, Kaohsiung Medical University Hospital, Kaohsiung Medical University, Kaohsiung, Taiwan; ^8^ Doctoral Degree Program of Toxicology, College of Pharmacy, Kaohsiung Medical University, Kaohsiung, Taiwan; ^9^ Department of Laboratory Medicine, School of Medicine, College of Medicine, Kaohsiung Medical University, Kaohsiung, Taiwan; ^10^ Ta-Kuo Clinic, Kaohsiung, Taiwan; ^11^ Department of Pediatrics, Kaohsiung Municipal Ta-Tung Hospital, Kaohsiung, Taiwan; ^12^ Drug Development and Value Creation Research Center, Kaohsiung Medical University, Kaohsiung, Taiwan; ^13^ Department of Superintendent, High Commissioner, Kaohsiung Municipal Siaogang Hospital, Kaohsiung, Taiwan; ^14^ Department of Nursing, Fooyin University, Kaohsiung, Taiwan; ^15^ Department of Gynecology and Obstetrics, E-Da Hospital, Kaohsiung, Taiwan; ^16^ School of Medicine, College of Medicine, I-Shou University, Kaohsiung, Taiwan; ^17^ Department of Pediatrics, Kaohsiung Medical University Hospital, Kaohsiung Medical University, Kaohsiung, Taiwan; ^18^ Department of Pediatrics, Kaohsiung Municipal Siaogang Hospital, Kaohsiung, Taiwan

**Keywords:** childhood allergy, aryl hydrocarbon receptor, Di(2-ethylhexyl) phthalate, flavonoids, IL-33

## Abstract

**Background:**

Few studies assess cord blood biomarkers to predict prenatal exposure to di(2-ethylhexyl) phthalate (DEHP) on the development of allergic diseases later in childhood. IL-33 has been indicated to play an important role in allergic diseases. We evaluated the association of prenatal DEHP exposure and IL-33 in cord blood on the development of allergic diseases. We also investigated the mechanism of DEHP in human lung epithelial cells and asthma animal models.

**Methods:**

66 pregnant women were recruited, and their children followed when they were aged 3 years. Maternal urinary DEHP metabolites were determined using liquid chromatography-electrospray-ionization-tandem mass spectrometry. The effect of DEHP on IL-33 production was investigated in human lung epithelial cells and club cell-specific aryl hydrocarbon receptor (AhR) deficiency mice. ELISA and RT-PCR, respectively, measured the IL-33 cytokine concentration and mRNA expression.

**Results:**

The concentrations of maternal urinary DEHP metabolites and serum IL-33 in cord blood with childhood allergy were significantly higher than those in the non-childhood allergy group. DEHP and MEHP could induce IL-33 production and reverse by AhR antagonist and flavonoids *in vitro*. Enhanced ovalbumin-induced IL-4 and IL-33 production in bronchoalveolar lavage fluid (BALF) by DEHP exposure and suppressed in club cell-specific AhR null mice. Kaempferol has significantly reversed the DEHP effect in the asthma animal model.

**Conclusions:**

Cord blood IL-33 level was correlated to childhood allergy and associated with maternal DEHP exposure. IL-33 might be a potential target to assess the development of DEHP-related childhood allergic disease. Flavonoids might be the natural antidotes for DEHP.

## 1 Introduction

Over the past several decades, the prevalence of asthma, allergic disease, and atopy has increased significantly worldwide (1). Endocrine-disrupting chemicals (EDCs) are commonly found in the environment. They are derived from industrial and agricultural sources, including pesticides, fungicides, insecticides, herbicides, and other chemicals used in the plastics industry. The increasing prevalence of both autoimmune and allergic diseases paralleled the increasing usage of plasticizers and the increasing levels of their derivatives as a form of EDCs in the environment (2). In a cross-sectional study, a combination of the presence of allergens and polyvinylchlorid (PVC) flooring was the strongest determinant of doctor-diagnosed asthma. The indoor dust concentration of DEHP in the bedroom is also a risk factor for asthma (3).

Interleukin (IL)-33 is a tissue-derived nuclear cytokine from the IL-1 family abundantly expressed in endothelial cells, epithelial cells, and fibroblast-like cells, which are important for the initiation and development of airway immune responses to environmental stimuli (4). IL−33 levels correlate with clinical asthma severity, and IL33 variants have been implicated in susceptibility to allergic rhinitis and the risk of asthma (5). An important role for IL-33 is activating type 2 innate lymphoid cells (ILC2s), which produce IL-5 and IL-13 to activate T-helper-2 cells and promote the persistence of airway eosinophilia in patients with severe asthma (6). A similar mechanism is likely to occur for IL−33 activity in patients with atopic dermatitis, who have elevated levels of IL−33 in the skin epidermis (7).

Phthalate substances used in manufacturing plastics are considered possible human carcinogens and have adverse effects through nuclear receptors, such as aryl hydrocarbon receptor (AhR) (8). AhR, a member of the periodic circadian protein (PER)–AHR nuclear translocator (ARNT)–single-minded protein (SIM) superfamily, is a ligand-activated transcription factor. It is expressed in most cell types, including immune cells, epithelial cells, and cells of the central nervous system. It sensed endogenous factors (redox potential) and exogenous factors (polyaromatic hydrocarbons and environmental toxins). After AhR activation, it translocated to the nucleus and controls the expression of target genes harboring AHR-responsive DNA elements (dioxin response elements, DREs) in their regulatory regions (9). Flavonoids are plant polyphenols and have attracted a great deal of attention because of their anticancer and anti-inflammatory activities (10). Flavonoids exert their functions and effects through the AhR signaling pathway and are antagonists of the binding effect (11).

Since both flavonoids and phthalates exert their effects through nuclear receptors, we hypothesized that flavonoids may be natural protectants against phthalates. However, the roles and relationships of IL-33 and maternal di(2-ethylhexyl) phthalate (DEHP) exposure in the pathogenesis of later-developed childhood allergies remain unclear. In the present study, we first revealed evidence of IL-33 in prenatal DEHP exposure-related childhood allergies. Additionally, we identified a protectant against DEHP-induced effects on the cytokine IL-33 in a lung epithelial cell model. We also tried to evaluate whether flavonoids could reverse the adverse effects of phthalates.

## 2 Materials and methods

### 2.1 Study population

This study subjects were from Taiwanese Maternal and Infant Cohort pilot study, which was approved by the Institutional Review Board of E-Da Hospital (EMRP35101N) and Kaohsiung Medical University Hospital (KMUHIRB-2012-11-02(I)). Written informed consent was obtained from all pregnant women in the study and on behalf of their children. The detailed study design and questionnaire are comprehensively described elsewhere (12). Children were evaluated at the age of 3 years to investigate allergic symptoms. Physician diagnosis of asthma, atopic dermatitis, or allergic rhinitis were made according to an operational description suggested by the Global Initiative for Asthma (GINA) guidelines and the Allergic Rhinitis and Its Impact on Asthma (ARIA) guidelines. The exclusion criteria for children included severe systemic diseases, such as congenital heart diseases, or autoimmune diseases.

### 2.2 Analysis of phthalate metabolites

Urinary DEHP metabolites and creatinine levels were measured in single urine sample from the pregnant women according to a modified method previously described (12). Creatinine-corrected adjustment for each individual was used to examine associations between the compound exposure and allergic diseases. The daily intake level of DEHP (∑DEHP) was estimated using a creatinine excretion-based model, and the detailed method was described in this cohort profile (13).

### 2.3 Cell preparation

Human bronchial epithelium (HBE) cells (American Type Culture Collection, Rockville, USA) were cultured in keratinocyte-SFM (Thermo Fisher Scientific, Waltham, USA) supplemented with 25 nM hydrocortisone and an 850 nM insulin solution (Sigma–Aldrich, St. Louis, USA). The A549 human lung carcinoma cell line (American Type Culture Collection) was cultured in MEM supplemented with 10% fetal bovine serum, 1% nonessential amino acids, 1% sodium pyruvate, 100 U/ml penicillin, and 100 μg/ml streptomycin. Cells were centrifuged, resuspended in fresh media, plated in 6-well plates at a density of 1 × 10^6^ cells/ml, and incubated for 24 h before use in experiments. The cells were treated with DEHP or mono-(2-ethylhexyl) phthalate (MEHP) (Sigma–Aldrich) at different concentrations (0.001-0.1 μM) or combined with house dust mites (HDM) extract (Greer Laboratories, Lenoir, USA), which experimental dose of HDM was referred to previous study (14) for 3 or 24 h for isolated RNA or protein measurement. Cells were pretreated with CH-223191 (AhR antagonist, Sigma–Aldrich), flavonoids (apigenin or kaempferol, Sigma–Aldrich), the histone acetyltransferase inhibitor, anacardic acid (AA, Sigma–Aldrich), or the methyltransferase inhibitor, 5’-deoxy-5’-(methylthio) adenosine (MTA, Sigma–Aldrich) 1 h before DEHP or MEHP treatment to identify involved receptors or signaling pathways. The experimental dose of phthalates, AhR antagonist, flavonoids and histone acetyltransferase/methyltransferase inhibitor were referred to our previous publication (15).

### 2.4 Quantitative real-time reverse transcription polymerase chain reaction

Total RNA and cDNA was performed as described in our previously published study (16). qRT–PCR was performed using SYBR Green PCR Master Mix on an ABI 7500 Real-Time PCR system. The qRT–PCR primers for *IL33* are as follows: forward: 5’-caaagaagtttgccccatgt, reverse: 5’-aaggcaaagcactccacagt according to the previous study (14). The mRNA expression levels were normalized to the cycle threshold value of the housekeeping gene *GAPDH* (forward: 5’-ccactcctccacctttgac, reverse: 5’-accctgttgctgtagcca). All reagents and kits were purchased from Thermo Fisher Scientific.

### 2.5 Chromatin immunoprecipitation assay

The ChIP assay was performed as described in our previously published study (16). Equal amounts of DNA from each sample were used to perform qRT–PCR to quantitate the amount of DNA using primers designed for the *IL33* promoter regions (forward: 5’-cagatctggagcagctgttc, reverse: 5’-aggccgtggtcactcatatt) according to the prediction from PROMO version 3.0.2 software. The relative amounts of the amplified products were normalized to the total input DNA amount in the samples.

### 2.6 Ovalbumin -sensitized and -challenged asthma models

The animal experiments followed the guidelines of the Animal Center of Kaohsiung Medical University and was approved by the Kaohsiung Medical University–Institutional Animal Care and Use Committee (IACUC-106054). We generated club cell-specific AhR KO mice (*Scgb1a1-Cre; AhR^flox/flox^*) as previously described (17). All mice, including wild-type (*Scgb1a1-Cre*) and *Scgb1a1-Cre; AhR^flox/flox^*, were maintained in a specific pathogen-free facility, randomly divided into four groups, and orally fed 0.01% DMSO group in corn oil, 25μg DEHP/kg body weight (DEHP group) in corn oil, 20 mg/kg body weight kaempferol (K group) or both DEHP and kaempferol (DEHP+K group) in corn oil (Sigma–Aldrich) every day. The mice were sensitized with 100 µg OVA by intraperitoneal injection on day 4. The mice were challenged with 3% aerosolized OVA over three successive days (days 15–17). The mice were sacrificed on day 18 for examinations of cytokine production and histology. The experimental concentration of DEHP was referred to DEHP daily intake in children in the indoor environment (18). We choose a dosage of kaempferol that could suppress allergic airway inflammation as previously described (19). The flow-chart of the treatment in experimental animal showed in **Supplementary Figure 1**.

### 2.7 Flow cytometry analysis

Bronchoalveolar lavage fluid (BALF) procedures was generated as described previously with a minor modification (17). Tracheae was cannulated and the lung was lavaged 2 times with 1 mL PBS (contain 1% FCS). Each fluid was centrifuged and the supernatant was rapidly frozen at −80°C. The cells in BALF were stained with CD11c-PE/Cy7 (N418), MHC class II-FITC (M5/114.15.2), anti-B220 (RA3–6B2; eBioscience, Thermo Fisher Scientific), CCR3-PE (83,101; R&D Systems, Minneapolis, USA), and CD3-APC (145–2C11; BD Biosciences, San Diego, USA) and antibodies and analyzed by flow cytometry (LSR II; BD Biosciences) (17). The gating strategy of immune cells in BALF was showed in **Supplementary Figure 2**.

### 2.8 Lung pathology

Lung samples were fixed in formaldehyde, embedded in paraffin, cut into 3 μm thick sections, stained with hematoxylin and eosin for general inflammation analyses, and observed by a light microscope (Axio Imager M1; Carl Zeiss, Oberkochen, Germany) (17).

### 2.9 Enzyme-linked immunosorbent assay (ELISA)

Concentrations of IL-4 and IL-33 in BALF or supernatant were determined by commercially ELISA systems using the protocol recommended by the manufacturer (R&D Systems).

### 2.10 Statistical analysis

Continuous variables were presented as the mean ± standard deviation (SD) were tabulated to describe the distribution. Pearson’s chi-square tests were used to evaluate the significance of differences in categorical variables between non-allergic groups and allergic groups. Independent sample t-test was used to examine the differences in total IgE or cord blood IgE between non-allergic groups and allergic groups; spearman correlation was used to examine the correlation between maternal urinary metabolite levels and cord blood cytokine levels in allergic groups. Statistical analysis was performed using SPSS (Version 20, IBM Company, Armonk, NY, USA) or GraphPad Prism (Version 5, Los Angeles, CA, USA). The bar graphs display the means ± standard deviation (SD). Statistical analysis was performed using SPSS (Version 20, IBM Company, Armonk, NY, USA) or GraphPad Prism (Version 5, Los Angeles, CA, USA). The Mann–Whitney U test was used to determine differences between control and experimental group. The one-way ANOVA with Bonferroni post-test was used to analysis the results of the animal data. A p value < 0.05 was considered to indicate a significant difference.

## 3 Results

The mean age of the 66 children who were followed up was 4.36 ± 0.59 years, and there were 38 males and 28 females. According to physician diagnosis, the children were stratified into the allergic or non-allergic groups. The demographic characteristics of the children and their mothers at baseline are presented in **Table 1**, and there was no significant difference in age, sex, or smoking exposure between these two groups.

**Table 1:** Demographic characteristics of 66 study mother and child.

	Non-allergic group (n=22)	Allergic group (n=44)	
**Mother’s information**			*P* value
Age, year (Mean ± SD)	30.7 ± 5.5	31.5 ± 3.7	0.521
Education			
≤ Senior high school	9 (40.9%)	9 (20.5%)	0.079
> Senior high school	13 (59.1%)	35 (79.5%)	
Smoking during pregnancy			
Yes	0 (0%)	0 (0%)	
No	21 (95.5%)	43 (97.7%)	
Missing	1 (4.5%)	1 (2.3%)	
Second-hand smoking during pregnancy			
Yes	5 (22.8%)	13 (29.5%)	0.592
No	16 (72.7%)	30 (68.2%)	
Missing	1 (4.5%)	1 (2.3%)	
Allergy-related history			
Yes	4 (18.2%)	17 (38.6%)	0.114
No	17 (77.3%)	27 (61.4%)	
Missing	1 (4.5%)	0 (0%)	
DEHP metabolites concentration			
MEHP, µg/g creatinine (mean ± SD)	2.53 ± 3.18	6.56 ± 8.84	0.009
MEOHP, µg/g creatinine (mean ± SD)	8.55 ± 4.58	17.28 ± 21.10	0.011
MEHHP, µg/g creatinine (mean ± SD)	10.01 ± 6.65	21.23 ± 25.35	0.008
MECPP, µg/g creatinine (mean ± SD)	14.53 ± 7.06	29.05 ± 33.68	0.008
MCMHP, µg/g creatinine (mean ± SD)	3.73 ± 1.72	6.98 ± 8.49	0.018
∑ DEHP, μg/kg _body weight_/day (mean ± SD)	2.87 ± 2.14	3.33 ± 2.83	0.5
**Child’s information**			*P* value
Age, year (Mean ± SD)	4.36 ± 0.68	4.36 ± 0.55	0.963
Gender			
Female	10 (45.5%)	18 (40.9%)	0.725
Male	12 (54.5%)	26 (59.1%)	
Gestational age at birth, weeks	38.8 ± 1.2	38.7 ± 1.3 ^a^	0.789
Delivery methods			
Normal spontaneous delivery	15 (68.2%)	28 (63.6%)	0.715
Caesarean section	7 (31.8%)	16 (36.4%)	
Second-hand smoking exposed			
Yes	9 (40.9%)	15 (34.1%)	0.628
No	11 (50.0%)	24 (54.5%)	
Missing	2 (9.1%)	5 (11.4%)	
Raise pets			
Yes	5 (22.8%)	6 (13.6%)	0.297
No	14 (63.6%)	34 (77.3%)	
Missing	3 (13.6%)	4 (9.1%)	
Cord blood total IgE, IU/mL (Mean ± SD)	0.58 ± 0.89 ^†^	1.22 ± 1.99 ^‡^	0.165
Total IgE, IU/mL (Mean ± SD)	189.51 ± 334.54 ^§^	302.13 ± 544.24 ^¶^	0.488

^†^1 missing data; ^‡^2 missing data; ^§^9 missing data; ^¶^6 missing data.

### 3.1 Serum IL-33 levels and maternal exposure to DEHP were related to the occurrence of allergies at 3 years old

**Figure 1:**
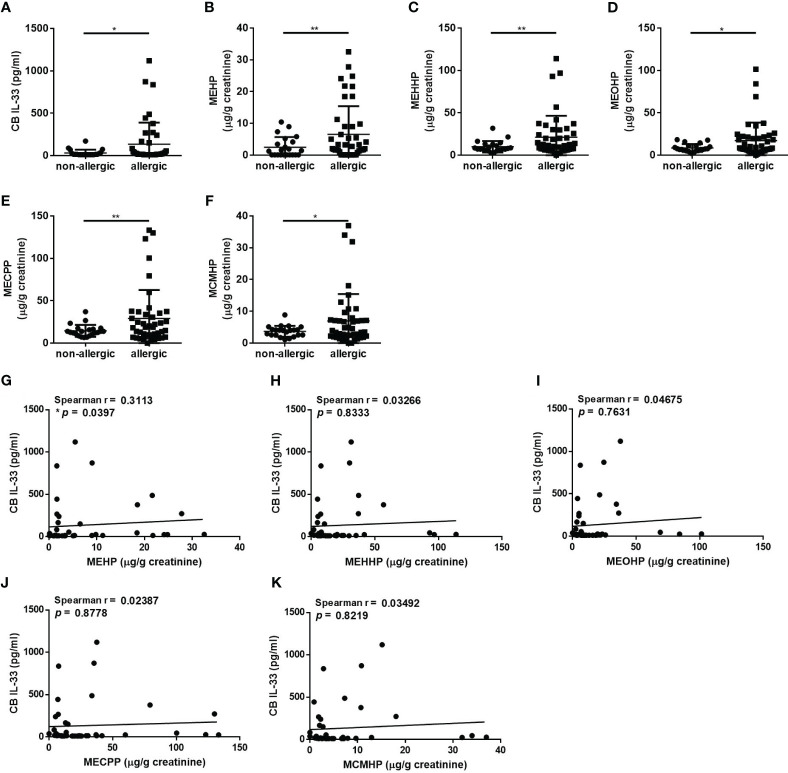
The relationships of maternal DEHP exposure and cord blood IL-33 levels with childhood allergy. The serum IL-33 concentration in cord blood (CB) was determined by ELISA. Maternal urinary DEHP metabolites were measured by liquid chromatography-electrospray ionization-tandem mass spectrometry (LC/ESI-MS/MS) and normalized to urinary creatinine levels. **(A)** Comparison of the difference in serum IL-33 levels between the allergic and non-allergic group groups. **(B-F)** Comparison of the maternal urinary mono-(2-ethylhexyl) phthalate (MEHP), mono(2-ethyl-5-hydroxyhexyl) phthalate (MEHHP), mono(2-ethyl-5-oxohexyl) phthalate (MEOHP), mono(2-ethyl-5-carboxypentyl) phthalate (MECPP), and mono(2-carboxymethylhexyl) phthalate (MCMHP) levels between the allergic and non-allergic group groups. **(G-K)** The correlation analysis between maternal urinary DEHP metabolite levels and CB IL-33 levels was assessed. *p < 0.05 and **p < 0.01. Data are shown as the mean ± SD.

IL-33 has recently been strongly recognized for its role in asthma and allergic diseases, the earliest manifestations of childhood allergy (5). We analyzed the cord blood (CB) IL-33 levels to examine the susceptibility of the newborn immune system to the potential adverse effects of *in utero* exposure to phthalates. The CB IL-33 levels in the allergic group were significantly higher than those in the non-allergic group (**Figure 1A**). We further investigated the levels of maternal urinary DEHP metabolites concentrations on the phenotype of allergic diseases. DEHP is rapidly metabolized to its monoester, MEHP, which is further metabolized by various hydroxylation and oxidation reactions to secondary metabolites, e.g., mono(2-ethyl-5-oxohexyl) phthalate (MEOHP), mono(2-ethyl-5-hydroxyhexyl) phthalate (MEHHP), mono(2-ethyl-5-carboxypentyl) phthalate (MECPP), and mono(2-carboxymethylhexyl) phthalate (MCMHP) (20). In particular, these metabolites appear suitable for assessing human DEHP exposure (biological monitoring) because they are not subject to external contamination. The concentrations of five common urinary DEHP metabolites in pregnant women were significantly higher in the allergic group than in the non-allergic group (**Figures 1B-F**). We next analyzed whether the CB IL-33 level was associated with maternal urinary DEHP metabolites level in the allergic group. The results showed a significantly positive correlation between MEHP and CB IL-33 (**Figure 1G**), but such a relationship was not shown for the other secondary metabolites (**Figures 1H-K**). We also observed whether the difference of CB IL-33 levels in different clusters including maternal allergic history, pets or not, second-hand smoking exposure during pregnancy, and the severity of allergic rhinitis in child. In the maternal allergic history cluster, the results showed the level of CB IL-33 was no difference in mother with or without allergic history. In the other clusters, the level of CB IL-33 also observed no difference between each two groups (**Supplementary Table 1**). We further used the geometric mean (geo mean) to divide samples into low or high groups (21) based on maternal urinary MEHP (geo mean: 1.944 µg/g creatinine) and CB IL-33 levels (geo mean: 39.34 pg/mL). We distinguished the children in allergic groups into four groups, group 1 (elevated CB IL-33 and diminished maternal urinary MEHP), group 2 (diminished CB IL-33 and diminished maternal urinary MEHP), group 3 (diminished CB IL-33 and heightened maternal urinary MEHP), and group 4 (elevated CB IL-33 and heightened maternal urinary MEHP). We want to investigate the potential dissimilarities in phenotypic expression among these four groups. In **Supplementary Table 2**, the maternal age, exposure to second-hand smoking during pregnancy, maternal allergic history, children’s age, gender, delivery methods, exposure to second-hand smoking, and raised pets showed no difference in these four groups. The prevalence of asthma, allergic rhinitis, and atopic dermatitis also showed no difference in these four groups. Otherwise, the secondary maternal urinary DEHP metabolites (MEOHP, MEHHP, MECPP, and MCMHP) were also elevated in group 3 and group 4. However, the CB total IgE and total IgE were also no different between these four groups. Even the total IgE in group 1 and 4 seemed higher than group 2 or group 3, but it was no statistical significance.

### 3.2 DEHP, MEHP and HDM extract induced IL-33 expression in A549 and HBE cells

**Figure 2:**
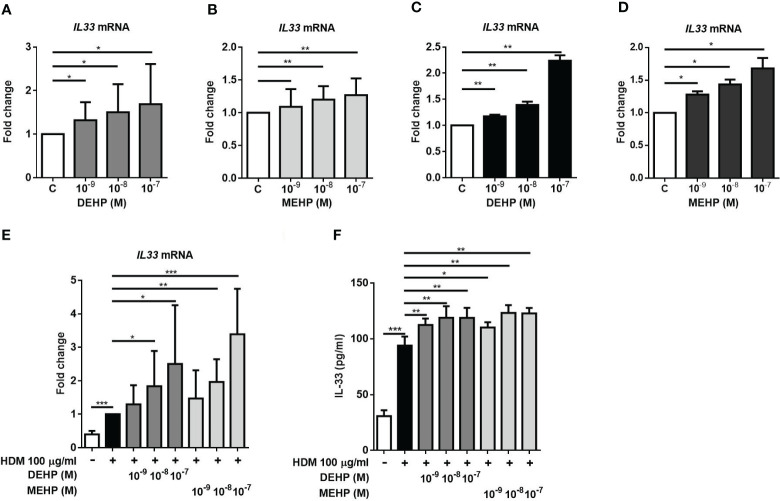
Effect of DEHP and its metabolite MEHP on IL-33 production in epithelial cells. A549 cells or HBE cells were incubated with solvent control (control), DEHP, or MEHP (0.001-0.1 μM) for 3 hrs. IL-33 mRNA expression was measured by real-time PCR in A549 cells **(A, B)** or HBE cells **(C, D)**. After pretreatment with DEHP or MEHP for 2 h, A549 cells were stimulated with house dust mites (HDM, 100 μg/ml) for an additional 3 h or an additional 24 h, and cytokines in the supernatant were analyzed. The IL-33 mRNA **(E)** and protein **(F)** levels in DEHP- or MEHP-treated A549 cells in response to exposure to HDM extract were assessed. *p < 0.05, **p < 0.01, and ***p < 0.001. Data are shown as the mean ± SD of 4 independent experiments.

Since the CB IL-33 levels were significantly higher in the allergic group and high MEHP group, to examine whether DEHP and its important metabolite MEHP affect IL-33 production in human lung and bronchial epithelial cells. As shown in **Figure 2**, DEHP or MEHP induced IL33 mRNA expression (**Figures 2A, B**) in a dose-dependent manner in A549 cells. In HBE cells, IL33 mRNA expression was also increased in a dose-dependent manner after DEHP or MEHP treatment (**Figures 2C, D**). We further used HDM (*Dermatophagoides pteronyssinus*) extract as a common allergen to evaluate the effect of DEHP or MEHP on IL-33 production. We observed that DEHP or MEHP treatment enhanced IL-33 mRNA expression (**Figure 2E**) and cytokine production (**Figure 2F**) in A549 cells. However, DEHP or MEHP alone did not induce IL-33 protein production (data not shown).

### 3.3 AhR antagonists and flavonoids suppress DEHP- and MEHP-induced IL-33 expression

**Figure 3:**
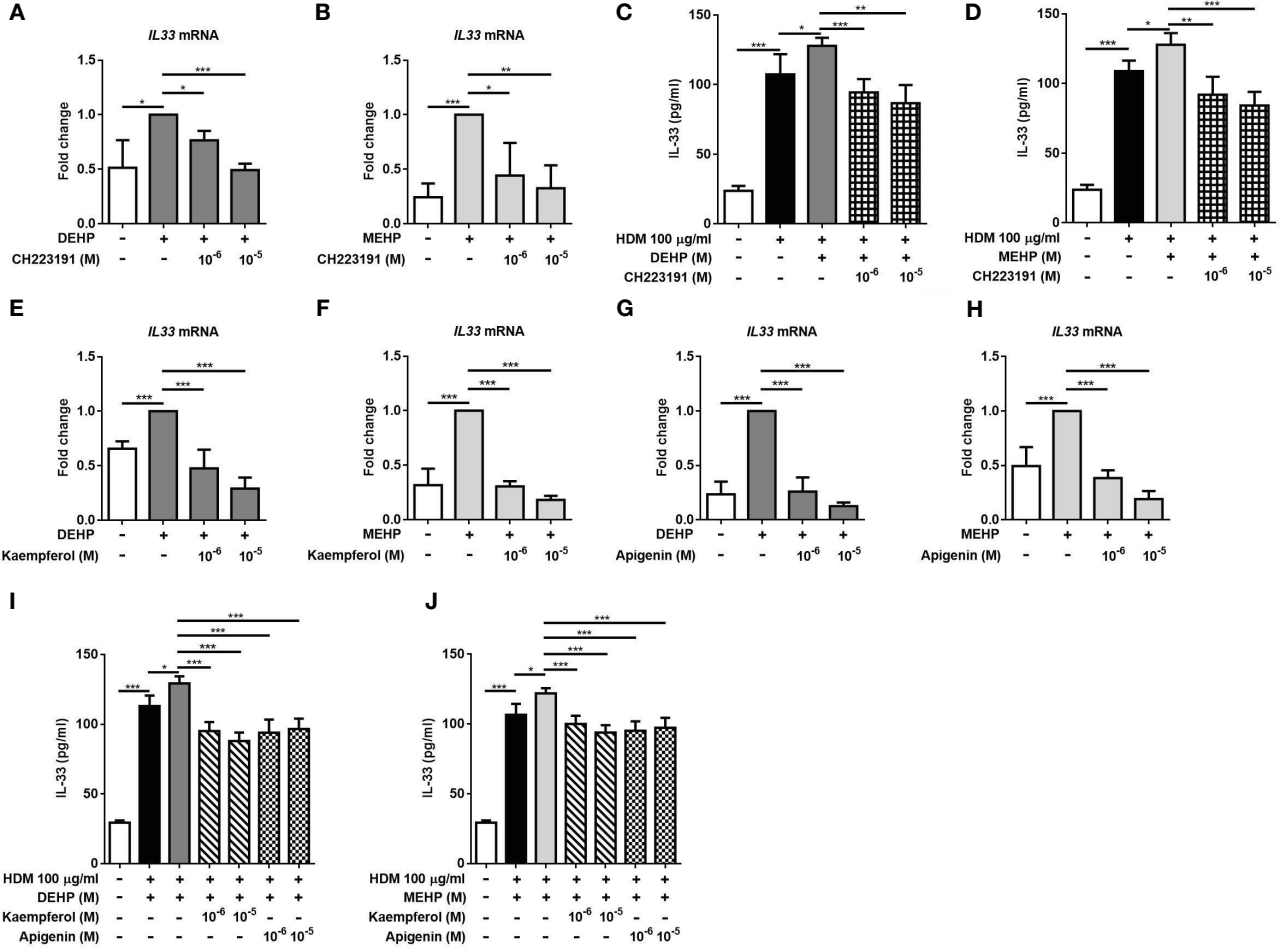
AhR antagonists and flavonoids affect DEHP- or MEHP-induced IL-33 expression. After pretreatment with an aryl hydrocarbon receptor (AhR) antagonist (CH223191; 1 or 10 μM) **(A, B)** or the flavonoids kaempferol **(C, D)** or apigenin **(E, F)** for 1 h, A549 cells were incubated with solvent control (control), DEHP, or MEHP (0.001-0.1 μM) for 3 h, and the level of IL-33 mRNA was measured. The cells were treated with the AhR antagonist **(G, H)** or the flavonoids **(I, J)** for 1 h and DEHP or MEHP (0.01 μM) for an additional 2 h and then stimulated with HDM extract (100 μg/ml) for an additional 24 (h) The IL-33 concentration in the supernatants was determined using ELISA. *p < 0.05, **p < 0.01, and ***p < 0.001. Data are shown as the mean ± SD of 4 independent experiments.

We further added an AhR antagonist to investigate the suppressive effect on DEHP- or MEHP-induced IL-33 production. Our results showed that the expression of IL33 mRNA induced by DEHP or MEHP stimulation were reversed by the AhR antagonist (**Figures 3A, B**). The increase in HDM-induced IL-33 production induced by DEHP or MEHP treatment was also reduced by the AhR antagonist (**Figures 3C, D**). These results suggested that the regulatory effects of phthalates on IL-33 might be mediated through nuclear AhR. Flavonoids have attracted great attention for their anticancer and anti-inflammatory activities (22). Therefore, we hypothesized that flavonoids might suppress the expression of IL-33 through nuclear AhR. The results showed that DEHP and MEHP-induced IL33 mRNA expression was suppressed by kaempferol and apigenin (**Figures 3E-H**). The increase in HDM-induced IL-33 production induced by DEHP or MEHP treatment was reduced by kaempferol and apigenin (**Figures 3I, J**). We also chose the AhR agonist, 2,3,7,8-tetrachlorodibenzodioxin (TCDD), which dose referred to the previous study (23), to observe whether the AhR was involved in MEHP-induced IL-33 expression. In **Supplementary Figure 3**, the expression of IL-33 mRNA was significantly increased by MEHP or TCDD treatment and suppressed by pretreatment with AhR antagonist or kaempferol. These results implied that the suppressive effect of flavonoids on IL-33 expression might be mediated through AhR.

### 3.4 DEHP and MEHP induced IL33 expression *via* histone modification

**Figure 4:**
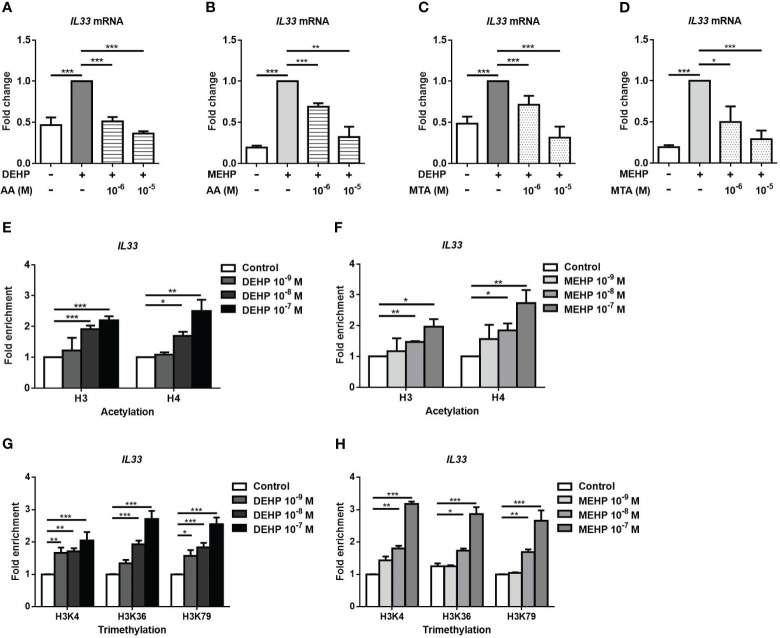
The effect of histone modification on DEHP- or MEHP-induced IL33 mRNA expression. After pretreatment with anacardic acid (AA) **(A, B)** or 5′-deoxy-5′-(methylthio) adenosine (MTA) **(C, D)** for 1 h, A549 cells were incubated with solvent control (control), DEHP or MEHP (0.001-0.1 μM) for 3 h, and the level of IL-33 mRNA was measured. **(E-H)** A549 cells were treated with MEHP at the indicated concentrations for 0.5 h and subjected to ChIP assays using antibodies against acetyl-H3, acetyl-H4, trimethyl-H3K4, trimethyl-H3K36, or trimethyl-H3K79. Purified DNA fragments were amplified for quantitative real-time PCR using primers to generate fragments within the IL33 promoter. The fold enrichment relative to the promoter was determined as the 2^-ΔΔCT^ value of DEHP- or MEHP-treated cells/the 2^-ΔΔCT^ value of vehicle-treated cells. *p < 0.05, **p < 0.01, and ***p < 0.001. Data are shown as the mean ± SD of 4 independent experiments.

Histone modifications are a component of epigenetic regulation and an important modulator of gene expression (24). We used AA and MTA to evaluate whether histone modifications were involved in the DEHP- or MEHP-induced increases in the expression of the IL33 mRNAs. The increased level of IL33 mRNA induced by DEHP or MEHP was decreased by AA (**Figures 4A, B**). Moreover, the DEHP- or MEHP-induced increases in IL33 mRNA expression were also suppressed by MTA (**Figures 4C, D**). Histone H3 and H4 acetylation in the IL33 promoter region were upregulated by DEHP or MEHP stimulation, especially at high doses (**Figures 4E, F**). Furthermore, histone H3K4, H3K36, and H3K79 trimethylation in the IL33 promoter region were also upregulated (**Figures 4G, H**). We also observed whether the AhR was involved in histone modification by MEHP stimulation. In **Supplementary Figure 4A**, the upregulated acetylation level of histone H3 and H4 by MEHP stimulation were reduced by AhR treatment. The MEHP-regulated tri-methyl H3K4, H3K36, and H3K79 were also downregulated by AhR treatment. These results show that DEHP- or MEHP-induced changes in the expression of the IL33 mRNAs are regulated by histone H3 and H4 acetylation and histone H3K4, H3K36, and H3K79 trimethylation *via* AhR.

### 3.5 Kaempferol reversed DEHP-enhanced IL-33 production and lung inflammation in OVA-sensitized and -challenged mice

**Figure 5:**
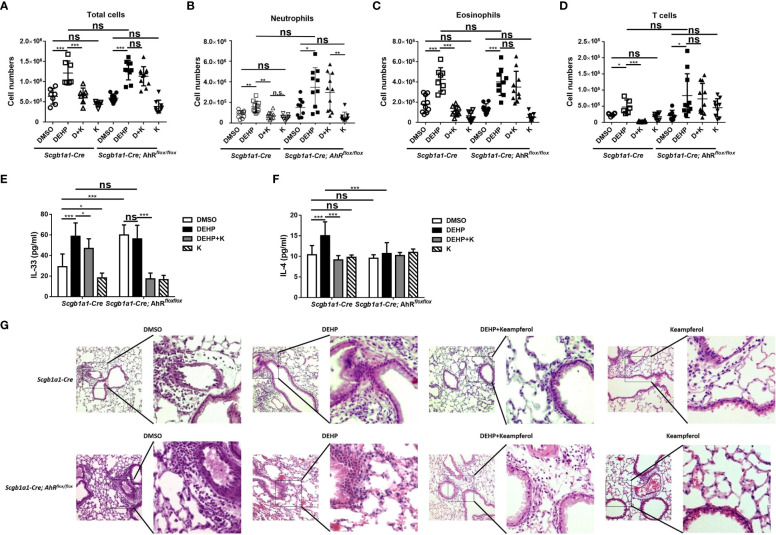
DEHP enhanced severity of ovalbumin (OVA)-induced lung inflammation through aryl hydrocarbon receptor, and reversed by kaempferol. **(A-D)** Cell subsets in bronchoalveolar lavage fluid (BALF) samples were identified using flow cytometry. The IL-4 **(E)** and IL-33 **(F)** levels in BALF samples from *Scgb1a1-Cre* or club cell-specific AhR-null mice (*Scgb1a1-Cre; AhR^flox/flox^*) were measured by ELISA. **(G)** H&E-stained lung sections of OVA-sensitized and -challenged mice. Representative photomicrographs (200×) of the lung sections are shown. *p < 0.05, **p < 0.01, and ***p < 0.001. Data are shown as the mean ± SD ns, not significant..

In *Scgb1a1-Cre* mice, the number of total cells in bronchoalveolar lavage fluid (BALF) in the DEHP injection group was significantly increased compared with that in the dimethyl sulfoxide (DMSO) group and was significantly reduced after co-feeding of kaempferol. However, the suppressed effect of kaempferol did not shown in *Scgb1a1-Cre; AhR^flox/flox^* mice (**Figure 5A**). The counts of neutrophils, eosinophils, and T cells in BALF showed similar patterns both in *Scgb1a1-Cre* and *Scgb1a1-Cre; AhR^flox/flox^* mice (**Figures 5B-D**). We also measured the production of the Th2 cytokines IL-4 and IL-33 in BALF. OVA-induced IL-4 and IL-33 production was significantly enhanced by DEHP and suppressed by kaempferol in *Scgb1a1-Cre* mice (**Figures 5E, F**, left side). We further observed the cytokines IL-33 and IL-4 in *Scgb1a1-Cre; AhR^flox/flox^* to assess the role of AhR in the DEHP response. In *Scgb1a1-Cre; AhR^flox/flox^* mice, the IL-33 and IL-4 concentrations were not different between the DMSO and DEHP groups, and IL-33 was significantly decreased by kaempferol (**Figures 5E, F**, right side). Histological staining showed that exposure to DEHP further enhanced lung inflammation in *Scgb1a1-Cre* mice but not in *Scgb1a1-Cre; AhR^flox/flox^* mice, and was alleviated by kaempferol (**Figure 5G**). The results suggested that DEHP enhances lung inflammation and the production of the IL-33 and IL-4 through AhR.

## 4 Discussion

Epidemiological studies have shown the relationships between phthalate exposure and allergic disease (2). The fetal period is critical for immune system development, and susceptibility to the adverse effects of environmental exposure may be particularly high during this period. During this time, exposure to EDCs can promote permanent, irreversible changes to the developing immune system and increase the risk of an allergic phenotype (25). Therefore, exploring *in utero* exposures that may predict these changes is critical to understanding the association of maternal phthalate exposure with childhood allergic disease development. Ashley-Martin et al. reported that maternal urinary MCPP levels were associated with elevated levels of both IL-33/thymic stromal lymphopoietin (TSLP) and immunoglobulin E (IgE) (26). These data suggested that IL-33 and TSLP are detectable in CB and may be correlated with maternal phthalate exposure. In a previous study, the authors fed pregnant Sprague–Dawley rats with DEHP from gestational day 14 until parturition and found that DEHP exposure promoted chronic systemic inflammation in offspring (27). In our previous study, we used the DEHP transmaternal asthma model to investigate the allergic immune response in offspring. In this model, only the F0 female mice were exposed directly to DEHP, while F1 generation and naïve male mice were not subjected to any oral treatment. OVA-induced airway inflammation was significantly enhanced in F1 offspring and also noted in the OVA-immunized F2 and F3 progenies from DEHP-exposed F0 female mice. We demonstrated that maternal DEHP exposure could affect the development of allergic inflammation in offspring (28). However, there is still a lack of biomarkers for predicting childhood atopy associated with maternal phthalate exposure. The present study found that human CB IL-33 levels were associated with maternal DEHP exposure-related childhood atopy development. To our knowledge, this is the first study to find IL-33 as a potential target that can be used to assess the effects of prenatal exposure to phthalates on the risk of developing childhood allergies. Moreover, we tried to find potential phenotypic expression differences in our study population (**Supplementary Table 2**). However, these four groups did not show a significant difference in phenotypic expression. The reason might be due to a small sample size in our study.

Otherwise, not only IL-33 but also TSLP contributed to inducing allergic inflammation. Huihui You et al. reported that DEHP could enhance TSLP production in BALF and lung tissue. When they gave anti-TSLP monoclonal antibody, the DEHP enhanced airway inflammation, Th2 cytokines (IL-4, IL-5 and IL-13) production, and airway hyperresponsiveness were reduced *via* neutralized TSLP (29).

Several studies have suggested that co-exposure to environmental factors other than allergens can change the risk of developing allergic sensitization. For example, prenatal exposure to allergens was associated with a greater risk of allergic sensitization, which was increased by exposure to nonvolatile pollutants (30). Diesel exhaust particles can also act as adjuvants to promote sensitization to allergens and enhance the Th2 response in human experiments (31). IL- 33, a tissue-derived nuclear cytokine, observed that endogenous IL- 33 accumulates in the nuclei of producing cells, such as epithelial cells and fibroblasts. When tissue injury, IL-33 full length (IL-33FL) was rapidly released from the nuclei and cleaved by proteases from environmental allergens and/or from inflammatory cells. After cleaving, the IL-33 generated hyperactive mature forms exhibiting 30-to-60-fold higher activity than IL-33FL. IL-33FL at high doses has biologically active but has little activity at low doses. Due to this characteristic, the IL-33 protein expression levels by DEHP or MEHP treatment alone might not be more obvious than the mRNA level. When combined with HDM stimulating resulted to the level of IL-33 protein or mRNA were significantly increased in the present study. HDM is one of the common allergens that result in allergic inflammation. In the previous publication, the *Dermatophagoides pteronyssinus* is the most common allergy that causes allergic inflammation in Taiwan. Therefore, to mimic the exposure that humans typically encounter in daily life and the finding in clinical in Taiwan, we observed the effect of DEHP or MEHP combined HDM, particularly *Dermatophagoides pteronyssinus*, on IL-33 production (32).

AhR is a ligand-activated transcription factor that has been described as being involved in cell biological processes, such as chemical detoxification and immunity/inflammation (33). The allergic rhinitis animal study found that serum IL-33 levels and nasal mucosal oxidative stress significantly increased in the OVA + DEHP group than those in the OVA group. The AhR protein and its mRNA expression were also significantly increased in the OVA + DEHP group than in the OVA group (34). A previous study showed that the expression of AhR and IL-10 production was increased by lipopolysaccharide (LPS) stimulation in RAW264.7 cells. After LPS treatment, peritoneal and bone marrow-derived macrophages from AhR knockout mice showed reduced IL-10 production. These data suggested that AhR is involved in the immune regulation of inflammatory macrophages (35). Recent publications indicated that AhR mediated the IL-33 secretion by 2,3,7,8-tetrachlorodibenzo-p-dioxin (TCDD) (23) and IL-4 treatment (36). We also found that effect of DEHP on lung inflammation and cytokines production were mediated by AhR. It suggested that AhR involved in the DEHP mediated immune response. However, our results showed that the IL-33 level was increased with DMSO treatment in *Scgb1a1-Cre; AhR^flox/flox^* mice. Our recent publication revealed that AhR is a critical sensor in club cells that maintains a homeostatic state under inflammatory conditions. AhR deficiency reduced CC10, and SP-D failed to induce cell renewal. The increased IL-33 levels in *Scgb1a1-Cre; AhR^flox/flox^* mice might reflect defective protective mechanisms of club cells (17). Flavonoids have been reported to induce anticancer and anti-inflammatory effects (37). Hsu et al. reported reported that didymin, one of the flavonoids, could suppress DBP−mediated cell migration and invasion (38). Our previous study showed that apigenin and kaempferol could reverse the suppressed effect of DEHP on type I IFN production (15). Another study also indicated that flavonoid kurarinone inhibited LPS-induced pro-inflammatory cytokines such as TNF-α production and increased anti-inflammatory cytokine IL-10 production *via* AhR (39). The present study found that apigenin and kaempferol could reverse DEHP- and MEHP-induced IL-33 expression. Therefore, flavonoids could be natural substances that protect against the effects of DEHP.

Few studies have indicated that EDC-induced inflammatory responses are regulated by epigenetic regulation (40). Our previous study showed that DEHP decreased type I IFN production through AhR and downregulated H3K4 trimethylation in the IRF7 gene promoter region in human plasmacytoid dendritic cells (15). As previous study, the IL-33 production was decreased after sublingual immunotherapy by upregulated H3K27 trimethylation in the IL33 promoter region (41). The present study showed that DEHP and MEHP can upregulate H3 and H4 acetylation and H3K4, H3K36, and H3K79 trimethylation in the IL33 gene promoter region. It suggested that histone modifications regulated IL-33 production and participated in the effect of DEHP. Several birth cohort studies have found an association between prenatal phthalate exposure and childhood allergy (25). However, there is still no strategies for preventing or combating maternal phthalate exposure. The present study indicated that IL-33 might be a potential target to assess the development of prenatal DEHP exposure-related childhood allergy. Since it is difficult to avoid using plasticizers related to childhood allergies associated with maternal phthalate exposure, we encourage the consumption of flavonoids to prevent phthalate-related immunotoxicity.

## Data availability statement

The raw data supporting the conclusions of this article will be made available by the authors, without undue reservation.

## Ethics statement

The studies involving human participants were reviewed and approved by The Institutional Review Boards of E-Da Hospital (EMRP35101N) and Kaohsiung Medical University Hospital (KMUHIRB-2012-11-02(I)). Written informed consent to participate in this study was provided by the participants’ legal guardian/next of kin. The animal study was reviewed and approved by The Institutional Animal Care and Use Committee of Kaohsiung Medical University (IACUC-106054).

## Author contributions

Conceptualization: M-LT, M-CF, F-CK and C-HH. Methodology: M-LT, S-HH and C-HH. Formal analysis: L-TW, C-HK, W-TL and Y-CL. Investigation: M-CF. Datacuration: L-TW, F-CK, S-HH, and Y-LH. Writing-original draft preparation: M-LT. Writing-reviewing and editing: M-CF, F-CK and C-HH. All authors reviewed the manuscript. All authors contributed to the article and approved the submitted version.
