# Supplementary material for: Di(2-ethylhexyl) phthalate mediates IL-33 production via aryl hydrocarbon receptor and is associated with childhood allergy development
Source: Front Immunol. 2023 Jul 21;14:1193647. doi: 10.3389/fimmu.2023.1193647 (PMC10401841; doi:10.3389/fimmu.2023.1193647)
Supplement: Supplementary file 1 [file DataSheet_1.docx]

**Supplementary Material and Methods**

**Quantitative real-time reverse transcription polymerase chain reaction (qRT–PCR)**

The A549 cells (American Type Culture Collection, Rockville, MD, USA) were cultured in MEM (GIBCO, St. Louis, MO, USA) with 10% fetal bovine serum, antibiotic antimycotic Solution, non-essential amino acids, and sodium pyruvate. The cells were pretreated AhR antagonist, CH223191 or kaempgerol 1 h fooled by MEHP (10^-8^ M) or AhR agonist, 2,3,7,8-tetrachlorodibenzodioxin (TCDD) treatment for an additional 3 h for mRNA expression analysis. qRT–PCR primers for *IL33* are as follows: forward: 5’-caaagaagtttgccccatgt, reverse: 5’-aaggcaaagcactccacagt according to the previous study(1). The mRNA expression levels were normalized to the cycle threshold value of the housekeeping gene *GAPDH* (forward: 5’-ccactcctccacctttgac, reverse: 5’-accctgttgctgtagcca).

**Statistical analysis**

Continuous variables were presented as the mean ± standard deviation (SD) were tabulated to describe the distribution. Kruskal-Wallis test followed by Dunnett's multiple comparison test for continuous variables, and Fisher's exact test for categorical variable were used to examine the differences between these four groups. Statistical analysis was performed using SPSS for Windows, version 20.0. (SPSS Inc., Chicago, Ill., USA). All p-values were two-sided and the significance was < 0.05.

**Supplementary Table 1.** The level of cord blood IL-33 in different clusters.

|  | No | Yes | *P* value |
| --- | --- | --- | --- |
| Maternal allergic history ^†^ | 89.48 ± 215.2^$^ | 125.5 ± 217.4^$^ | 0.1439 |
| Second-hand smoking during pregnancy^‡^ | 116.7 ± 247.5^$^ | 64.31 ± 97.09^$^ | 0.7572 |
| Raise pets^¶^ | 101.3 ± 196.5^$^ | 28.63 ± 46.36^$^ | 0.1364 |
|  | Mild  (n = 30) | Moderate – Sever  (n = 13) | *P* value |
| Severity of allergic rhinitis in child | 169.4 ± 292.3^$^ | 66.18 ± 121.2^$^ | 0.4033 |
| ^$^: mean ± SD, ^†^: 1 missing data; ^‡^: 2 missing data; ^¶^: 7 missing data | | | |

Supplementary Table 2. Demographic characteristics of 4 groups in allergic child.

|  | **Group 1**  **N = 7** | | **Group 2**  **N=14** | | **Group 3**  **N=14** | | **Group 4**  **N=9** | | ***p* value** | |
| --- | --- | --- | --- | --- | --- | --- | --- | --- | --- | --- |
| **Mother’s information** |  | |  | |  | |  | |  | |
| Age, year (mean ± SD) | 31.91 ± 3.35 | | 31.33 ± 4.14 | | 30.68 ± 3.76 | | 32.63 ± 3.46 | | 0.667 | |
| Second-hand smoking during pregnancy |  | |  | |  | |  | |  | |
| Yes | 3 (42.9 %) | | 5 (35.7 %) | | 3 (21.4 %) | | 2 (22.2 %) | | 0.718 | |
| No | 4 (57.1 %) | | 9 (64.3 %) | | 11 (78.6 %) | | 6 (66.7 %) | |  |  |
| Missing | 0 | | 0 | | 0 | | 1 (11.1 %) | |  |  |
| Allergy-related history |  | |  | |  | |  | |  | |
| Yes | 4 (57.1 %) | | 4 (28.6 %) | | 6 (42.9 %) | | 3 (33.3 %) | | 0.610 | |
| No | 3 (42.9 %) | | 10 (71.4 %) | | 8 (57.1 %) | | 6 (66.7 %) | |  |  |
| DEHP metabolites concentration |  | |  | |  | |  | |  | |
| MEHP, µg/g creatinine (mean ± SD) | 1.24 ± 0.77 | | 0.56 ± 0.64 | | 11.32 ± 10.15^#,$^ | | 12.63 ± 9.11^#,$^ | |  | |
| MEOHP, µg/g creatinine (mean ± SD) | 4.14 ± 2.36 | | 10.39 ± 6.18 | | 24.41 ± 30.23^#^ | | 27.14 ± 20.26^#^ | |  | |
| MEHHP, µg/g creatinine (mean ± SD) | 4.75 ± 2.79 | | 13.81 ± 9.62 | | 28.41 ± 34.33^#^ | | 34.42 ±27.63^#^ | |  | |
| MECPP, µg/g creatinine (mean ± SD) | 6.35 ± 3.92 | | 16.67 ± 8.20 | | 39.33 ± 40.80^#^ | | 49.99 ± 43.31^#^ | |  | |
| MCMHP, µg/g creatinine (mean ± SD) | 1.28 ± 1.01 | | 4.46 ± 2.63 | | 9.37 ± 11.11^#^ | | 11.60 ± 10.01^#^ | |  | |
| ∑ DEHP, μg/kg _body weight_/day (mean ± SD) | 1.75 ± 1.21 | | 2.40 ± 1.41 | | 4.20 ± 3.62 | | 4.65 ± 3.23 | |  | |
| **Child’s information** | |  | |  | |  | |  | |  |
| Age, year | | 4.16 ± 0.41 | | 4.59 ± 0.60 | | 4.21 ± 0.32 | | 4.38 ± 0.77 | | 0.222 |
| Gender | |  | |  | |  | |  | |  |
| Female | | 2 (28.6 %) | | 8 (57.1 %) | | 5 (35.7 %) | | 3 (33.3 %) | | 0.505 |
| Male | | 5 (71.4 %) | | 6 (42.9 %) | | 9 (64.3 %) | | 6 (66.7 %) | |  |
| Delivery methods | |  | |  | |  | |  | |  |
| Normal spontaneous delivery | | 6 (85.7 %) | | 8 (57.1 %) | | 10 (71.4 %) | | 4 (44.4 %) | | 0.317 |
| Caesarean section | | 1 (14.3 %) | | 6 (42.9 %) | | 4 (28.6 %) | | 5 (55.6 %) | |  |

Supplementary Table 2 cont. Demographic characteristics of 4 groups in allergic child.

|  | **Group 1**  **N = 7** | **Group 2**  **N=14** | **Group 3**  **N=14** | **Group 4**  **N=9** | ***p* value** |
| --- | --- | --- | --- | --- | --- |
| **Child’s information** |  |  |  |  |  |
| Second-hand smoking exposed |  |  |  |  |  |
| Yes | 2 (28.6 %) | 5 (35.7 %) | 4 (28.6 %) | 4 (44.4 %) | 0.637 |
| No | 5 (71.4 %) | 7 (50.0 %) | 9 (64.3 %) | 3 (33.3 %) |  |
| Missing | 0 | 2 (14.3 %) | 1 (7.1 %) | 2 (22.2 %) |  |
| Raise pets |  |  |  |  |  |
| Yes | 1 (14.3 %) | 3 (21.4 %) | 2 (14.3 %) | 0 | 0.592 |
| No | 6 (85.7 %) | 10 (71.4 %) | 11 (78.6 %) | 7 (77.8 %) |  |
| Missing | 0 (0 %) | 1 (7.1 %) | 1 (7.1 %) | 2 (22.2 %) |  |
| Asthma | 2 (28.6 %) | 2 (14.3 %) | 1 (7.1 %) | 1 (11.1 %) | 0.598 |
| Allergic rhinitis | 7 (100 %) | 13 (92.9 %) | 14 (100 %) | 9 (100 %) | 0.213 |
| Mild | 5 (71.4 %) | 8 (61.5 %) | 10 (71.4 %) | 7 (77.8 %) | 0.869 |
| Moderate-Severe | 2 (28.6 %) | 5 (38.5 %) | 4 (28.6 %) | 2 (22.2 %) |  |
| Atopic dermatitis | 4 (57.1 %) | 5 (35.7 %) | 2 (14.3 %) | 1 (11.1 %) | 0.110 |
| CB IL-33 levels, pg/mL (mean ± SD) | 268.51 ± 289.59^$,^* | 14.58 ± 3.35 | 17.34 ± 6.28 | 402.89 ± 370.02^$,^* |  |
| CB total IgE, IU/mL (mean ± SD) | 1.53 ± 2.80^†^ | 1.13 ± 2.15^†^ | 0.94 ± 1.04 | 1.57 ± 2.51 |  |
| Total IgE, IU/mL (mean ± SD) | 791.00 ± 1090.86 | 89.81 ± 208.37^‡^ | 263.98 ± 257.02^‡^ | 242.61 ± 244.60^‡^ |  |
| Group1: high levels of CB IL-33 and low levels of maternal urinary MEHP; Group 2: low levels of CB IL-33 and maternal urinary MEHP; Group 3: low levels of CB IL-33 and high levels of maternal urinary MEHP; Group 4: high levels of CB IL-33 and maternal urinary MEHP; CB: cord blood ; ^†^ : 1 missing data; ^‡^: 2 missing data; ^#^: significant difference compared with group 1; ^$^: significant difference compared with group 2; *: significant difference compared with group 3. | | | | | |

**Supplementary Figure 1.** The schedule of OVA-induced lung inflammation in mice


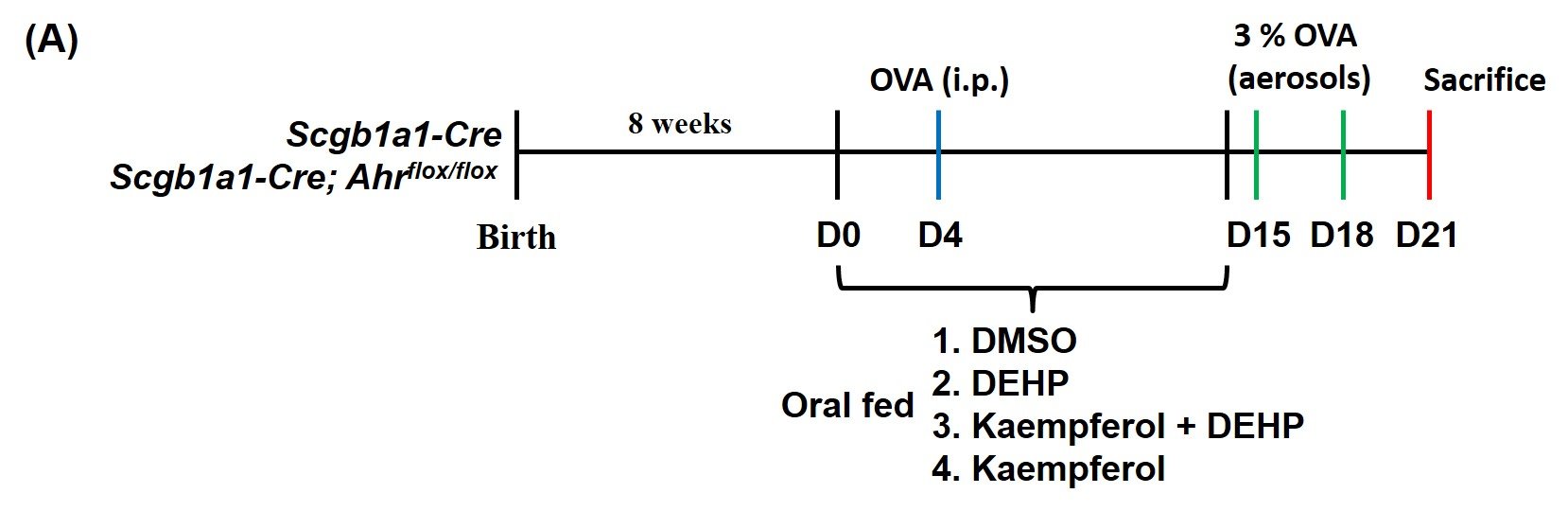


1. Experimental protocol of OVA-induced lung inflammation, in which mice were sacrificed 3 days after the last antigen challenge, and lung tissues and BALFs were collected for analysis.

**Supplementary Figure 2.** The gating strategy of immune cells in BALF by flow cytometry.


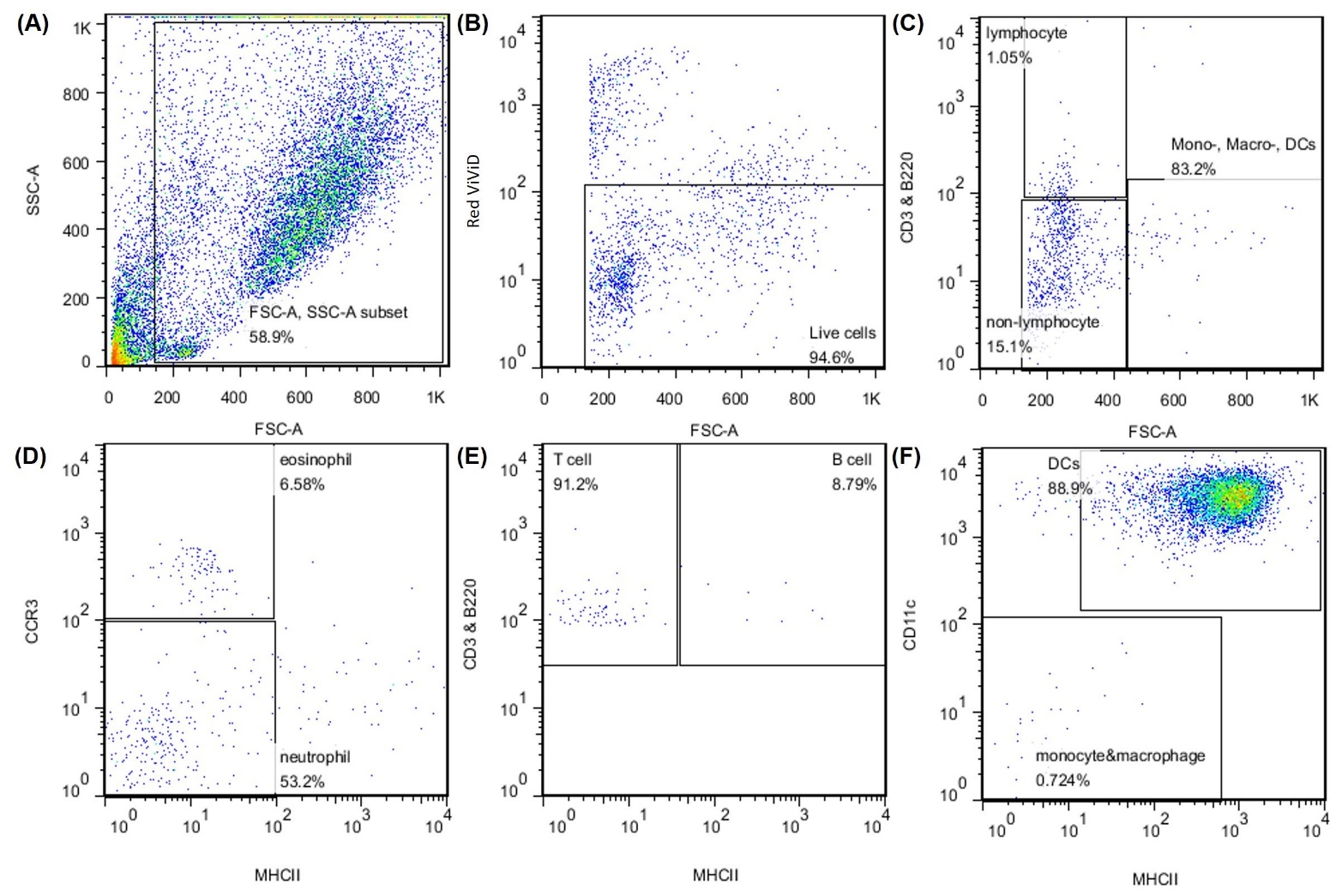


(B) Live cells were gated on a forward scatter (FSC)/Red ViViD plot. (C) Live cells were then further gated to determine lymphocyte, non-lymphocyte, and monocyte/macrophages/dendritic cells by CD3 and B220/FSC plot. (D) Non-lymphocyte were further gated to determine eosinophils (CCR3^+^ MHCII^-^) and neutrophils (CCR3^-^ MHCII^-^). (E) Lymphocyte were further gated to determine T cells (CD3/B220^+^ MHCII^-^) and B cells (CCR3^+^ MHCII^+^). (F) Monocyte/macrophages/dendritic cells were further gated to determine the monocyte/macrophages group or dendritic cells by CD11b.

**Supplementary Figure 3.** AhR antagonists and kaempferol affect MEHP- or TCDD-induced IL-33 expression.


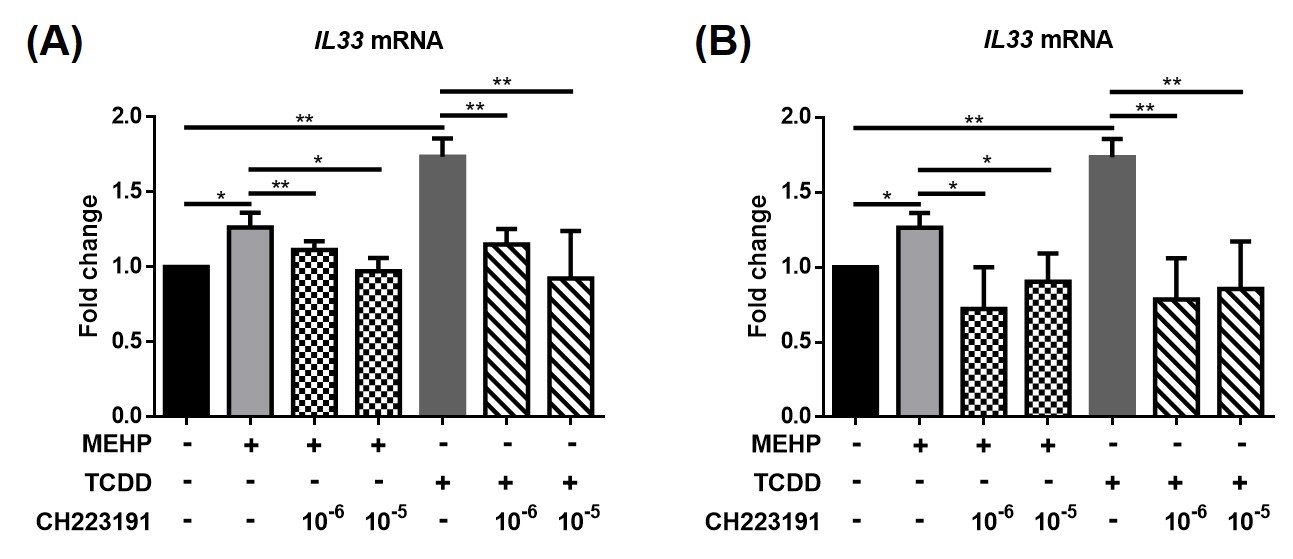


After pretreatment with an aryl hydrocarbon receptor (AhR) antagonist (CH223191; 1 or 10 μM) (A) or kaempferol (B) for 1 h, A549 cells were incubated with solvent control (control), MEHP (10^-8^ M) for 3 h, and the level of IL-33 mRNA was measured. * p < 0.05, ** p < 0.01, and *** p < 0.001. Data are shown as the mean ± SD of 3 independent experiments.

**Supplementary Figure 4.** The effect of histone modification on MEHP-induced IL-33 expression via AhR.


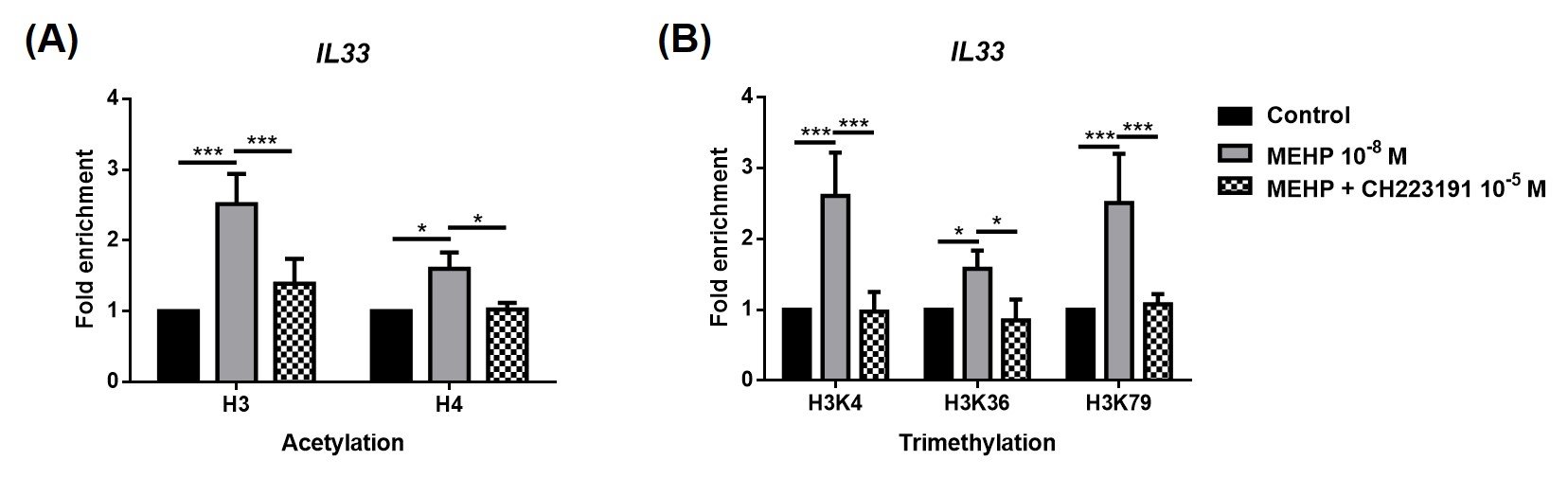


After pretreatment with CH223191 for 0.5 h, A549 cells were treated with MEHP (10^-8^ M) for 0.5 h and subjected to ChIP assays using antibodies against (A) acetyl-H3, acetyl-H4, (B) trimethyl-H3K4, trimethyl-H3K36, or trimethyl-H3K79. Purified DNA fragments were amplified for quantitative real-time PCR using primers to generate fragments within the IL33 promoter. The fold enrichment relative to the promoter was determined as the 2^-ΔΔCT^ value of DEHP- or MEHP-treated cells / the 2^-ΔΔCT^ value of vehicle-treated cells. * p < 0.05 and *** p < 0.001. Data are shown as the mean ± SD of 3 independent experiments.

Reference

1. Hristova M, Habibovic A, Veith C, Janssen-Heininger YM, Dixon AE, Geiszt M, et al. Airway epithelial dual oxidase 1 mediates allergen-induced IL-33 secretion and activation of type 2 immune responses. *J Allergy Clin Immunol*. 2016;137(5):1545-56 e11 <https://doi.org/10.1016/j.jaci.2015.10.003>
